# Appraisal of clinical practice guidelines for the management of attention deficit hyperactivity disorder (ADHD) using the AGREE II Instrument: A systematic review

**DOI:** 10.1371/journal.pone.0219239

**Published:** 2019-07-05

**Authors:** Yasser Sami Amer, Haya Faisal Al-Joudi, Jeremy L. Varnham, Fahad A. Bashiri, Muddathir Hamad Hamad, Saleh M. Al Salehi, Hadeel Fakhri Daghash, Turki Homod Albatti, on behalf of The Saudi ADHD Society

**Affiliations:** 1 CPG Unit, Quality Management Department, King Saud University Medical City, Riyadh, Saudi Arabia; 2 Pediatrics Department, King Khalid University Hospital, King Saud University Medical City, Riyadh, Saudi Arabia; 3 Research Chair for Evidence-Based Health Care and Knowledge Translation, Deanship of Scientific Research, King Saud University, Riyadh, Saudi Arabia; 4 Alexandria Center for Evidence-Based Clinical Practice Guidelines, Alexandria University, Alexandria, Egypt; 5 Department of Neurosciences, King Faisal Specialist Hospital and Research Center, Riyadh, Saudi Arabia; 6 Saudi ADHD Society, Riyadh, Saudi Arabia; 7 School of Psychology, University of East London, London, United Kingdom; 8 Division of Neurology, Department of Pediatrics, College of Medicine, King Saud University Medical City, King Saud University, Riyadh, Saudi Arabia; 9 Child Development Center, King Abdullah Bin Abdulaziz University Hospital, Princess Noura Bint AbdulRahman University, Riyadh, Saudi Arabia; 10 Ada’a Program, Assistant Deputyship for Hospital Services, Ministry of Health, Riyadh, Saudi Arabia; 11 Department of Psychiatry, King Saud University Medical City, King Saud University, Riyadh, Saudi Arabia; South-Central University for Nationalities, CHINA

## Abstract

**Background and objective:**

High quality evidence-based clinical practice guidelines (CPGs) have a major impact on the appropriate diagnosis and management and positive outcomes. The evidence-based healthcare for patients with attention deficit hyperactive disorder (ADHD) is challenging. The objective of this study was to appraise the quality of published CPGs for ADHD.

**Methods:**

A systematic review was conducted for ADHD CPGs using CPG databases, DynaMed, PubMed, and Google Scholar. The quality of each included CPG was appraised by three independent appraisers using the Appraisal of Guidelines for Research & Evaluation II (AGREE II) instrument.

**Results:**

Six CPGs were critically reviewed. The AGREE II standardized domain scores revealed variation between the quality of these CPGs with the National Institute of Health and Care Excellence (NICE), University of Michigan Health System, and American Academy of Pediatrics CPGs as the top three. Overall, the recommendations for management of ADHD were similar in these CPGs.

**Conclusions:**

Reporting of CPG development is often poorly documented. Guideline development groups should aim to follow the AGREE II criteria to improve the standards and quality of CPGs. The NICE CPG showed the best quality. Embedding the AGREE II appraisal of CPGs in the training and education of healthcare providers is recommended.

The protocol for this study was published in PROSPERO (International prospective register of systematic reviews). Link: http://www.crd.york.ac.uk/PROSPERO/display_record.php?ID=CRD42017078712 and is additionally available from protocols.io. Link: https://dx.doi.org/10.17504/protocols.io.q27dyhn.

## Introduction

Attention deficit hyperactivity disorder [1,2] or Attention-Deficit/ Hyperactivity Disorder [3,4] (ADHD), is a chronic neurodevelopmental disorder characterized by developmentally inappropriate levels of hyperactivity-impulsivity and/or inattention [1–9]. ADHD is clinically and genetically heterogeneous with multiple possible etiologies and frequent neuropsychiatric comorbidities [10,11]. ADHD is highly prevalent in 5–6% of children and in 3.8–4.4% of adults [12].

Clinical practice guidelines (CPGs) summarize the best available evidence and provide guidance for healthcare providers during their daily practice. CPGs can support the knowledge-to-action cycle effectively if they were developed using a systematic and rigorous methodology. Published evidence has revealed that CPGs can improve patient outcomes, patient experience, and quality and safety in healthcare [13].

In 2011, the Health and Medicine Division (HMD) of the American National Academies, formerly the Institute of Medicine (IOM), published its eight criteria of trustworthy CPGs, Clinical Practice Guidelines We Can Trust [14]. Since then, many sets of standards or criteria for high quality CPGs have been published or updated, including the Guidelines International Network’s [15], the GIN-McMaster Checklist [16], and the AGREE II Reporting Checklist [17], based upon the AGREE II Instrument’s 23 criteria. These standards have helped in shaping the development process and methodologies of CPGs worldwide [18].

Two systematic reviews of CPG appraisal tools have included a total of 64 tools [19,20]; these revealed that the AGREE II Instrument was the only tool that had a validated scoring system, as well as already being widely adopted. It has proven to become the international gold standard for quality assessment and development of CPGs, being cited more than 746 times between 2013–2018 [21].

A brief review of literature on the utilization of AGREE II for ADHD CPGs revealed two uses: One was restricted to psychopharmacological management of ADHD [22], and the other was conducted as part of a Master’s thesis in Pediatrics at Alexandria University [23]. The primary objective of this study is to provide a comprehensive, easily accessible, and updated assessment of the quality of available CPGs pertaining to ADHD diagnosis and management, using the gold standard instrument, AGREE II; CPGs included were published between 2012 and 2019, following the publication of the HDM and G-I-N CPG standards. Earlier published CPGs in general were found to be of variable quality and poor compliance with available methodological standards at that time [24,25]

## Methods

The protocol for this study was published in PROSPERO (International prospective register of systematic reviews). Link: http://www.crd.york.ac.uk/PROSPERO/display_record.php?ID=CRD42017078712 [26] and is also available from Protocols.io. Link: https://dx.doi.org/10.17504/protocols.io.q27dyhn [25].

### Eligibility criteria

Criteria for including CPGs were: (1) Evidence-based CPGs (i.e. with a clear description of the development methodology); (2) English language; (3) original source CPGs (de novo developed); (4) both national and international CPGs; (5) published between January 1, 2012 and July 1, 2017 (the search was further extended till June 15, 2019); (6) published by an organization or group authorship in a CPG database or peer-reviewed journal. Only the most current version of each source CPG was included whether in the format of a full CPG document retrieved from the developing organization’s official website or in the form of a full-text publication that was authored by the CPG development group.

We excluded CPGs that were published earlier than 2012, written in non-English language, presented as consensus or expert-based statements or CPGs, adapted from other source CPG(s), or that had single authorship. Relevant publications summarizing or reporting implementation of the included CPGs by different authors were not considered for this CPG appraisal.

### Information resources (identification of ADHD CPGs)

We used literature searches of bibliographic databases (Medline/PubMed and Google Scholar), EBSCO DynaMed Plus (US), and CPG databases: American Agency for Healthcare Research and Quality’s (AHRQ) National Guideline Clearinghouse (US), Guidelines International Network (GIN), Scottish Intercollegiate Guidelines Network (SIGN; UK), National Institute of Health and Care Excellence (NICE; UK), and the Australian National Health and Medical Research Council (NHMRC). We also searched databases of national and international societies specializing in fields related to ADHD (e.g. American Psychiatric Association, European Psychiatric Association).

### Search, Screen, and Study Selection

Keywords used included “*attention-deficit/hyperactivity disorder”* or “*ADHD*,” and “*guideline*,” “*practice guideline*,” “*clinical practice guideline*,” “*practice parameter*,” “*guidance*,” or “*recommendations*” [26–28].

The PubMed search strategy included "attention deficit hyperactivity disorder"[tiab] OR "ADHD"[tiab]. Filters activated: Guideline, Publication date from 2012/01/01 to 2018/06/30 (extended to 2019/6/15), Humans.

Additionally, we used the PIPOH (Patient Population, Interventions, Professionals, Outcomes, and Healthcare Setting) checklist [18,28] to further assist in the inclusion and exclusion process. The following is a description of the characteristics derived from and used via PIPOH: Our patient population (P) was children and adults being assessed for or with a diagnosis of ADHD. Interventions (I) included diagnosis (complaints of the parent, teacher, or adolescent, history and physical examination, psychological tools, investigations, comorbidities) and treatment (pharmacological treatment, psychological and behavioral interventions, adverse effects of treatments, treatment of adverse effects, monitoring, special cases, complementary medicine, and transition of care from childhood to adulthood). Type of professionals (P) included physicians (e.g. psychiatrists, pediatricians, and neurologists), clinical psychologists, pharmacists, nurses, dieticians, occupational therapists, and social workers. Major outcomes (O) included ADHD symptom severity, academic performance, functional status, side effects of stimulant medications, and quality of life. Healthcare setting (H) included primary, secondary, and tertiary care settings addressing assessment, treatment, and management of ADHD.

Two reviewers (YA, JV) independently screened titles and abstracts of retrieved CPGs and articles meeting the inclusion criteria. The screening was rechecked by three other reviewers (TA, FB, MH). Disagreements were resolved by further discussions with the entire group after retrieval and review of the full CPG documents or full-text articles, including links to any available supplemental documents or web pages. We repeated our search before the final manuscript re-submission in June 2019 based on the pre-publication peer review to identify any new eligible CPGs.

### Assessment of CPGs using the AGREE II Instrument

The AGREE II Instrument (www.agreetrust.org) consists of 23 items or questions organized in six domains including scope and purpose (items 1–3), stakeholder involvement (items 4–6), rigor of development (items 7–14), clarity of presentation (items 15–17), applicability (items 18–21), and editorial independence (items 22–23). Each item or question is scored on a Likert scale from one to seven, where 1 = strongly disagree and 7 = strongly agree. The AGREE II assessment was conducted by using the “My AGREE PLUS” online tool developed by the AGREE Enterprise. My AGREE PLUS supports the AGREE II assessment process by creating a CPG “appraisal group” for each CPG, compiling and calculating the items’ scores into domain scores, and generating the final reports. My AGREE PLUS users are required to complete a free registration process before starting the AGREE online assessment for a given CPG. Each CPG appraisal group is handled by a “coordinator” who registers group’s details, invites assessors, reviews data, and generates the final AGREE II reports. Two separate reports can be generated from My AGREE PLUS once the CPG group assessment is completed: One for the “ratings” (i.e. individual item scores and standardized domain scores) and another for the “comments.” Additionally, the AGREE website provides online audiovisual training resources for using the AGREE II Instrument, as well as videos describing different functionalities of the My AGREE PLUS online platform.

Seven AGREE II assessors were selected with a wide range of clinical expertise (a child psychiatrist, two pediatric neurologists, a developmental pediatrician, a clinical neuropsychologist, a clinical pharmacist, and a general pediatrician and CPGs methodologist). At the outset of this project, AGREE II capacity building was conducted for the assessors by the expert methodologist through training and hands-on sessions in the concepts and standards of CPGs, and using the instrument. Each reviewer independently scored his/her assigned CPGs. Each one of the included CPGs was independently appraised by three reviewers: two clinicians and a methodologist.

All assessors reviewed the full CPG document, in addition to any supplementary documents or links to online pages related to the guideline’s methodology or implementation tools. For each item, AGREE assessors were asked to record the rationale for their scores in the comment section. Differences between assessors’ scores were resolved by asking those who had provided outlying scores to re-assess after discussion with the group. The disagreements were mainly observed in questions highly related to the CPG development methodology (i.e. questions 7–14 of domain 3) and implementation (especially question 18 of domain 5). The percentage of preliminary disagreements in some CPGs was 9 per 23 questions (39%) but were less in subsequently appraised CPGs with the rising learning and understanding curve for utilizing the AGREE II criteria for quality assessment. The standardized AGREE domain scores (ranging from 0 to 100%) were automatically calculated by My AGREE PLUS following the equations provided by the AGREE II User’s Manual.

A cut-off point of 60% for each AGREE standardized domain score was agreed upon by the reviewers, with more weight emphasized on the scores of domains three and five to facilitate the final assessment of the reporting quality of CPGs. Similar categorization of domains was recently reported and published [13,29,30].

An additional validation of the six CPGs for inclusion of systematic reviews with or without meta-analyses in their evidence-base and the frequency and percentage of Cochrane systematic reviews among these reviews was conducted.

Moreover, we checked whether the Grading of Recommendations Assessment, Development and Evaluation (short GRADE) methodology was utilized for the CPG development process as several CPG developing organizations are increasingly shifting to using the GRADE (e.g. World Health Organization, NICE, SIGN, NHMRC, etc.) [31–34]. The GRADE is a method of assessing the certainty in evidence (i.e. quality of evidence or confidence in effect estimates) and the strength of recommendations in health care. It has important implications for summarizing evidence for systematic reviews, health technology assessments, and CPGs as well as other decision makers [35,36].

## Results

### Identification of ADHD CPGs

The results of the search were summarized in S1 Fig. The initial list of 30 retrieved CPGs was reviewed, discussed and filtered by the assessors. Six recent ADHD CPGs complying with the identified PIPOH and inclusion criteria were eligible. These CPGs were developed by the American Academy of Pediatrics (AAP) in 2012, the University of Michigan Health System (UMHS) in 2012, the National Institute of Health and Care Excellence (NICE) in 2016 (updated in 2018), the National Health Medical Research Center (NHMRC) in 2013, the Canadian ADHD Resource Alliance (CADDRA) in 2014 (updated in 2018), and the Singapore Ministry of Health (SMOH) in 2014 [37–42].

An updated search and screen was conducted for ADHD Source CPGs in June 2019 using the same aforementioned information resources and criteria. This repeated search did not reveal any eligible CPG that needed to be added to the previous AGREE appraisal. Though excluded, several recent CPGs or relevant online material were worthwhile to mention due to the national and/or international impact of their publishing organizations.

Examples of these (with reasons for exclusion) include (i) the Interdisciplinary Evidence- and Consensus-based Guideline “ADHD in Children, Young People and Adults” June 2018 by the Association of the Scientific Medical Societies in Germany (AWMF) Online (in German) [43]; (ii) CPG on Therapeutic Interventions in ADHD 2017 by the Working group of the Clinical Practice Guideline on Therapeutic Interventions in ADHD, Ministry of Health, Social Services and Equality, Health Sciences Institute in Aragon (IACS) (in Spanish) [44]; (iii) the Updated European Consensus Statement on diagnosis and treatment of adult ADHD 2019 by the European Network Adult ADHD (ENAA) (Consensus statement) [45], (iv) British Association for Psychopharmacology’s (BAP) CPG for the pharmacological management of ADHD 2014 (Consensus statement) [46], and (v) the Centers for Disease Control and Prevention endorsed and posted the AAP treatment recommendations in their official website as of September 2018 (adopted AAP 2011 CPG) [47].

A number of online resources for the professionals and public summarized and discussed the recommendations of some these CPGs and relevant documents for example DynaMed Plus (updated in May 2019) [48,49], the World Federation ADHD Guide (2019) [50], and the ADHD Institute (updated in March 2019) [51] but without using a formal CPG appraisal tool like the AGREE II Instrument [19,20].

### Key characteristics of CPGs

Tables 1 and 2 demonstrate characteristics of all eligible CPGs. The eligible CPGs included five national CPGs (AAP, NICE, NHMRC, CADDRA, and SMOH) and one local CPG (UMHS). Two CPGs were developed by US-based organizations (n = 2, 33%), followed by one CPG (n = 1, 17%) developed by each of a Canadian-based, UK-based, Australian-based, and Singaporean-based organization respectively. The six included CPGs were developed by governmental bodies (n = 3, 50%), medical specialty society (n = 2, 33%), and healthcare improvement and CPG developer organizations (n = 2, 33%).

**Table 1:** Characteristics of the ADHD clinical practice guidelines (general).

Title	Year of publication	Country	Level of development	Organization (short name)	Total number of references
American Academy of Pediatrics (AAP) CPG on diagnosis, evaluation, and treatment of ADHD in children and adolescents (37)	2012	United States	National	AAP	70
Canadian ADHD Resource Alliance (CADDRA)-Canadian ADHD CPGs (CAP-Guidelines) 3^rd^ Edition (updated in 4^th^ edition). (39)	2015 (updated 2018)	Canada	National	CADDRA	264
National Health and Medical Research Council (NHMRC) Clinical Practice Points on diagnosis, assessment, and management of ADHD in children and adolescents (40)	2014	Australia	National	NHMRC	112
National Institute for Health and Care Excellence (NICE) ADHD CPG (42)	2016 (updated 2018)	United Kingdom	National	NICE	2941
Singapore Ministry of Health (SMOH) Guideline on ADHD (41)	2013	Singapore	National	SMOH	250
University of Michigan Health System ADHD (UMHS) (38)	2013	USA	Local	UMHS	14

**Table 2:** Characteristics of the ADHD clinical practice guidelines (clinical content/ options of care).

Clinical content/ Options of care	AAP (37)	CADDRA (39)	NHMRC (40)	NICE (42)	SMOH (41)	UMHS (38)
Diagnosis and Assessment						
1. Parent/ Teacher/ Patient (adolescent) complaints	Y	Y	Y	Y	Y	Y
2. Clinical picture	Y	N	Y	Y	N	Y
3. Psychological Tools	Y	Y	N	N	N	Y
4. Differential diagnosis	Y	Y	N	Y	Y	Y
5. Investigations	N	Y	N	N	Y	Y
6. Treatment						
7. Psychological and Behavioral interventions (PBI)	Y	Y	Y	Y	Y	Y
8. Pharmacological treatment	Y	Y	Y	Y	Y	Y
9. Treatment of adverse effects of pharmacological treatment	Y	Y	Y	Y	N	Y
10. Comorbidities	Y	Y	Y	Y	Y	Y
11. Monitoring	Y	Y	Y	Y	Y	Y
12. Special cases	Y	N	N	N	N	Y
13. Complementary medicine	Y	N	Y	N	Y	Y
14. Transition of care from childhood to adulthood	Y	N	Y	Y	Y	N
Y = Yes (included); N = No (not included)						

### Reporting the quality of ADHD CPGs

The AGREE II standardized domain scores were summarized in Table 3.

**Table 3:** AGREE II domain-standardized scores (ratings) for CPGs on management of ADHD.

ADHD CPGs (reference)/ AGREE II Domains-standardized scores	AAP (37)	CADDRA (39)	NICE (42)	NHMRC (40)	SMOH (41)	UMHS (38)
**Domain 1. Scope and Purpose** **Items 1–3:** Objectives; Health question(s); Population (patients, public, etc.).	80%	74%	100%	72%	37%	91%
**Domain 2. Stakeholder Involvement** **Items 4–6:** Group Membership; Target population preferences and views; Target users.	67%	50%	96%	76%	59%	43%
**Domain 3. Rigour of development** **Items 7–14:** Search methods; Evidence selection criteria; Strengths and limitations of the evidence; Formulation of recommendations; Consideration of benefits and harms; Link between recommendations and evidence; External review; Updating procedure.	66%	35%	93%	53%	47%	60%
**Domain 4. Clarity and presentation** **Items 15–17:** Specific and unambiguous recommendations; Management options; Identifiable key recommendations	76%	63%	89%	65%	83%	81%
**Domain 5. Applicability** **Items 18–21:** Facilitators and barriers to application; Implementation advice/ tools; Resource implications; Monitoring/ auditing criteria	64%	35%	92%	29%	69%	69%
**Domain 6. Editorial independence** **Items 22, 23:** Funding body; Competing interests	75%	78%	92%	67%	28%	69%
**Overall Assessment 1** (Overall quality)	56%	67%	100%	56%	50%	72%
**Overall Assessment 2** (Recommend the CPG for use)	*Yes-1*, *Yes with modifications-2*, *No-0*	*Yes-1*, *Yes with modifications-2*, *No-0*	*Yes-1*, *Yes with modifications-2*, *No-0*	*Yes-0*, *Yes with modifications-3*, *No-0*	*Yes-0*, *Yes with modifications-2*, *No-1*	*Yes-2*, *Yes with modifications-1*, *No-0*
**AGREE II Assessors**	*HA*, *TA*, *YA*	*SA*, *TA*, *YA*	*FB*, *MH*, *YA*	*HD*, *TA*, *YA*	*FB*, *HD*, *YA*	*HA*, *SA*, *YA*

American Academy of Pediatrics (AAP), the University of Michigan Health System (UMHS), National Institute of Health and Care Excellence (NICE), the National Health Medical Research Center (NHMRC), the Canadian ADHD Resource Alliance (CADDRA), and the Singapore Ministry of Health (SMOH), Clinical Practice Guidelines (CPGs), AGREE II (Appraisal of Guidelines for Research and Evaluation Instrument Version II)

#### Domain 1: Scope and purpose

The AGREE II standardized score for domain 1 ranged from 37% to 100%. Scores of all CPGs were greater than 60% in domain 1 except the SMOH CPG, in which the limited description of overall objectives, health questions, and patient populations resulted in a lower score. The two CPGs scoring more than 90% were NICE and UMHS.

#### Domain 2: Stakeholder involvement

The AGREE II standardized domain scores for domain 2 ranged from 43% to 96%. Scores of all CPGs were greater than 60% in domain 1 except the SMOH, CADDRA, and UMHS CPGs. The lack of adequate descriptions of patient preferences or target users resulted in the low scores for these CPGs. Only the NICE CPG scored more than 90%.

#### Domain 3: Rigor of development

The AGREE II standardized scores for domain 3 ranged from 35% to 93%. Three CPGs received scores greater than or equal to 60%: NICE (93%), AAP (66%), and UMHS (60%). The rest (NHMRC, CADDRA, and SMOH) received less than 60% in domain 3. Comprehensive search methods and strategy, evidence selection criteria, strengths and limitations of the evidence (evidence tables), detailed process of formulation of recommendations, discussion of the process of trade-off between risks and benefits, process of external review, and details of the updating process were the most common weaknesses among the NHMRC, CADDRA, and SMOH CPGs.

#### Domain 4: Clarity of presentation

The AGREE II standardized scores for domain 4 ranged from 63% to 89%. Scores of all CPGs were greater than 60% in domain 4. This domain was well-addressed in all included CPGs, where recommendations were specific, unambiguous, and easily identifiable in all CPGs. Three CPGs scored more than 80% (NICE, SMOH, and UMHS).

#### Domain 5: Applicability

The AGREE II standardized scores for domain 5 ranged from 29% to 92%. Scores of all CPGs were greater than 60% in domain 5 except CPGs for CADDRA and NHMRC, where facilitators, barriers, monitoring and auditing criteria, resource implications, and formal cost-analyses were not addressed. NICE CPG received the highest score, being the only guideline that received a score above 90%.

#### Domain 6: Editorial independence

The AGREE II standardized scores for domain 6 ranged from 28% to 92%. Scores of all CPGs were greater than 60% except the SMOH CPG.

#### Overall assessment

The AGREE II standardized domain scores for overall assessment ranged from 50% to 100%. All CPGs scored greater than 60% in the first overall assessment, except AAP, NHMRC and SMOH. Overall the NICE CPG received the highest scores on all six AGREE II domains, in addition to the highest score in the first overall assessment; it was the only CPG that received a score of 100%.

#### Recommending the CPGs for use in practice

The second (overall) assessment, pertaining to the overall recommendation for using the given CPG in clinical practice, revealed a variation between this score and the individual scores of domains in each CPG. This could be illustrated in the NICE CPG where this second overall assessment did not reflect a similarly high score as the scores received in the other six domains and the first overall assessment (i.e. in it, two assessors recommended NICE CPG for use with modifications, and one recommended it for use without modifications). A similar result was noted in the assessment of the AAP and CADDRA despite lower scores in other domains. UMHS was recommended for use by two appraisers. Nevertheless, there was an observed overall consistency in the recommendations of ADHD management throughout the included CPGs despite the variable strengths and weaknesses in each CPG according to the AGREE II criteria. This included diagnosing ADHD using the DSM-5 criteria, identifying comorbidities, initiation of the psycho-social or psycho-behavioral treatment, different management plans according to the age group, and stepwise approach of the pharmacological treatment with psycho-stimulants as the first-line.

All included CPGs cited systematic reviews and meta-analyses in their references list. The largest number of systematic reviews was observed in the evidence-base of the CPGs from NICE (N = 67), SMOH (N = 17), NHMRC (N = 14), CADDRA (N = 7), AAP (N = 2), and UMHS (N = 1) in descending order. Cochrane systematic reviews were only included in three CPGs: NICE (n = 19, 28%), NHMRC (n = 5, 36%), and SMOH (4, 24%). Moreover, two Cochrane systematic reviews were mentioned in the text of the UMHS CPG but were not cited in the references section and henceforth were considered not reported (0) (S2 Table). Overall, the lines of management of ADHD were similar in these CPGs (S3 Table).

## Discussion

The aim of this systematic review was to explore the quality of and critically appraise recently published evidence-based CPGs for the management of ADHD in all age groups [26]. An additional purpose was to assist clinicians and CPG groups in identifying high-quality and trustworthy evidence-based CPGs for ADHD using the AGREE II criteria.

Internationally accepted standards and appraisal tools for evidence-based CPGs recommend the transparent reporting of the CPG development process. This process includes; (i) selection of the health topic, (ii) composition of the CPG development group, (iii) key health questions, (iv) scope of the CPG, (v) systematic evidence review and decision-making process, (vi) formulation and articulation of CPG recommendations, ratings of evidence and recommendations, and evidence-to-recommendations links, (vii) implementation considerations and tools, (viii) peer review and stakeholder consultations, (ix) CPG expiration and updating, (x) financial support and sponsoring organization, and (xi) management of conflicts of interest [14–18]. Our appraisal, conducted using the AGREE II Instrument, highlighted several areas for improvement in the methodological rigor of the ADHD CPGs included for critical appraisal. The ADHD CPGs had several gaps in their Rigor of Development (Domain 3), which is the largest (and the core) AGREE II domain and in their Applicability (Domain 5) as well. Highlighting the importance of these two domains has been suggested [52]. There was consistency in ADHD recommendations despite variable evidence-bases. This consistency may reflect consensus in the healthcare community towards management of ADHD, despite the absence of a strong evidence-base in some CPGs. The AGREE II instrument has undergone several updates and improvements. Some of the shortcomings of the AGREE II instrument has been addressed in a recently developed tool entitled ‘AGREE-REX’ (Recommendation EXcellence) that addresses clinical credibility and implementability of the CPG recommendations. This new tool has been validated and is currently being refined before being shared publicly [53].

To the best of our knowledge, this is the first study to systematically evaluate the quality of recently published CPGs for management of ADHD in all age groups using the complete AGREE II instrument. The ADHD CPG review by the Canadian Agency for Drugs and Technologies in Health, even though limited to pharmacological treatment, is highly consistent with our findings [19]. It listed the AAP, and SMOH as the most rigorous ADHD CPGs. The aforementioned Alexandria University thesis reviewed 4 ADHD CPGs identified at the time of that study (viz. NICE 2008, AAP 2011, SIGN 2009, and ICSI 2010), with similar findings for the NICE and AAP CPGs as this appraisal [23]. Andrade et al systematically reviewed CPGs for the assessment, prevention and treatment of disruptive behavior (including ADHD, oppositional defiant disorder, conduct disorder and aggression) in children and youth using the AGREE II Instrument. It priotitized three AGREE II domians, viz. domains 2,3, and 6, to classify CPGs [54]. Despite being more broad in the scope of the review in terms of diagnosis (i.e. disruptive behavior) and more specific in terms of the age group (i.e. children and youth), it revealed overall similar results including selecting domain 3(rigor of development) as a key domain for filtering CPGs and displaying NICE as a superior ADHD CPG [54]. Moreover, Andrade chose to use different cutoffs for quality ratings (viz. 50% for minimum and 70% for maximum) [54].

Our review showed that only one ADHD CPG applied the GRADE methodology to appraise the quality of evidence (NICE) [31]. The NICE CPG development methodology is based on internationally recognized CPG standards like the AGREE II criteria, the Guideline Implementability Appraisal tool, in addition to primary methodological research and evaluation conducted by NICE. It includes transparent and clear health questions, search strategy, selection criteria for evidence, critical appraisal of clinical and economic evidence, consultation and validation process, and a noted component for implementation considerations and tools [31]. The NHMRC announced on its website that it started developing CPGs using GRADE in 2016 which followed the publication date of its most recent ADHD CPG (2012) [33]. All six CPGs under study included cross-referenced systematic reviews but only three CPGs included Cochrane reviews (NICE, NHMRC, and SMOH) despite increased production of Cochrane reviews. Currently, the total number of ADHD-related systematic review protocols registered in the PROSPERO database is 417, which comprises 337 ongoing reviews, 35 completed but not published including this review under study, 39 completed and published, one review is ongoing update, and two reviews that were discontinued [55]. Four Cochrane reviews on; (i) cognitive-behavioral interventions for attention deficit hyperactivity disorder (ADHD) in adults, (ii) methylphenidate for attention deficit hyperactivity disorder (ADHD) in children and adolescents—assessment of adverse events in non-randomised studies; (iii) Pharmacological treatment for attention deficit hyperactivity disorder (ADHD) in children with comorbid tic disorders; and (iv) Amphetamines for attention deficit hyperactivity disorder (ADHD) in adults in addition to 9 protocols were published after the publication of the NICE CPG in 16^th^ of March 2018 [56–59]. Despite the utilization of systematic reviews in the included ADHD CPGs, an area for improvement remains regarding the utilization of high-quality systematic reviews like Cochrane reviews in these CPGs. Similar recommendations were reported by Vale et al [60].

Furthermore, other reviews were published for ADHD but none have utilized a validated tool such as the AGREE II Instrument except the review by Siexas et al. that utilized the first version of the AGREE Instrument [61–64]. This appraisal was also conducted by a multidisciplinary team and an expert methodologist, which adds a layer of strength to the assessment.

Moreover, an additional implication for practice is to encourage healthcare providers caring for patients with ADHD to adopt principles of ‘Evidence-Based’ rather than ‘Eminence-Based’ Healthcare in their daily practice through training and education on CPG standards and appraisal tools [65–68]

One limitation to utilizing the AGREE II instrument is that it does not comprehensively critically appraise other important items included in the GRADE methodology for CPG development (e.g. risk of bias, precision, consistency, directness, and publication bias).The selection of 60% as a cut-off point for standard domain scores is another potential limitation as the original AGREE II does not mandate such a cut-off but similar studies have used it previously [13]. Other raters may choose different cut-offs [54]

Another limitation of our review was the exclusion of Non-English CPGs from our set of appraised CPGs despite the existence of Dutch, Finnish, Norwegian, German, and Spanish ADHD CPGs. Similar exclusion criteria were selected in published AGREE appraisals for CPGs [64,69,70]

The results of this appraisal can be used as a main component of a CPG development or adaptation project for the management of ADHD. Furthermore, it highlights the importance of inclusion of the AGREE II Instrument as a part of the capacity building for clinicians to guide them during the identification and adoption of CPGs for use in their daily practice.

In conclusion, The AGREE II assessment of the six included ADHD CPGs revealed methodological shortcomings in several domains. We recommend several areas for improvement for future CPGs, using the AGREE II criteria and the NICE CPG as a model. This critical appraisal illustrates the importance of regular quality assessment of CPGs by clinicians to ensure the transparency and rigor of the CPG development process and the evidence-base management of patients with ADHD.

## Supporting information

S1 Fig PRISMA flow diagram. Systematically searching and selecting the clinical practice guidelines for management of ADHD.
(DOC)

S1 Protocol (PDF)

S1 Table PRISMA checklist. (DOC)

S2 Table Mapping of ADHD CPGs against systematic reviews, meta-analyses and the utilization of the GRADE method. (DOCX)

S3 Table Key recommendations of included ADHD CPGs. (DOCX)
