# Supplementary material for: Appraisal of clinical practice guidelines for the management of attention deficit hyperactivity disorder (ADHD) using the AGREE II Instrument: A systematic review
Source: PLoS One. 2019 Jul 5;14(7):e0219239. doi: 10.1371/journal.pone.0219239 (PMC6611626; doi:10.1371/journal.pone.0219239)
Supplement: S1 Fig — Systematically searching and selecting the clinical practice guidelines for management of ADHD. (DOC) [file pone.0219239.s001.doc]

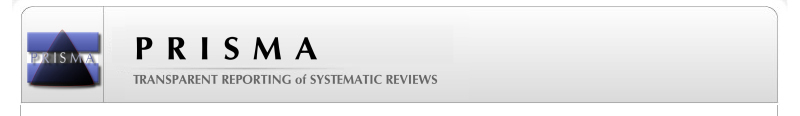
**S1 Fig. PRISMA 2009 Flow Diagram**

Systematically searching and selecting the clinical practice guidelines for management of ADHD

**Screening**

**Included**

**Eligibility**

**Identification**

Records after duplicates removed
*(n = 30)*

Records identified through CPG databases searching (AHRQ-NGC, DynaMed, G-I-N, SIGN, NICE, NHMRC, societies) *(n = 39)*

Additional records identified through other bibliographic sources (PubMed, Google Scholar) *(n = 9)*

Records screened

*(n = 30)*

Records excluded with reasons

*(n =22):* not matching PIPOH (8); not matching eligibility criteria (13); not CPGs (1)

Full-text articles assessed for eligibility by focus group discussions and more detailed review *(n = 8)*

Full-text articles excluded, with reasons *(n = 2)*

Studies included for quality assessment *(qualitative and quantitative synthesis)*

By the AGREE II Instrument

*(n = 6)*
