# Supplementary material for: Appraisal of clinical practice guidelines for the management of attention deficit hyperactivity disorder (ADHD) using the AGREE II Instrument: A systematic review
Source: PLoS One. 2019 Jul 5;14(7):e0219239. doi: 10.1371/journal.pone.0219239 (PMC6611626; doi:10.1371/journal.pone.0219239)
Supplement: S1 Protocol — (PDF) [file pone.0219239.s002.pdf]

Appraisal of clinical practice guidelines for the management of attention deficit hyperactivity disorder (ADHD) using the AGREE II Instrument: a systematic review

Turki Albatti, Saleh Al Salehi, Fahad Bashiri, Muddathir Hamad, Haya Al-Joudi, Hadeel Daghash, Jeremy Varnham, Yasser Amer

Citation

Turki Albatti, Saleh Al Salehi, Fahad Bashiri, Muddathir Hamad, Haya Al-Joudi, Hadeel Daghash, Jeremy Varnham, Yasser Amer. Appraisal of clinical practice guidelines for the management of attention deficit hyperactivity disorder (ADHD) using the AGREE II Instrument: a systematic review. PROSPERO 2017 CRD42017078712 Available from:
http://www.crd.york.ac.uk/PROSPERO/display_record.php?ID=CRD42017078712

Review question

The aim is to explore the quality of and critically appraise recently published evidence-based clinical practice guidelines for the management of Attention Deficit Hyperactivity Disorder (ADHD)

Searches

Data Sources include:-
• Guidelines International Network http://www.g-i-n.net/library/international-guidelines-library
• National Guidelines Clearinghouse http://www.guidelines.gov
• EBSCO - DynaMed Plus https://dynamed.ebscohost.com/
• National Institute for Health and Clinical Excellence http://www.nice.org.uk/
• Scottish Intercollegiate Guidelines Network http://www.sign.ac.uk/index.html
• US National Library of Medicine, National Institutes of Health (MEDLINE/ PubMed)
http://www.ncbi.nlm.nih.gov/PubMed (for CPGs published as full-text articles)
• Google Scholar (free) http://scholar.google.com/ (for CPGs published as full-text articles) • Plus CPGs published by national and international specialized scientific societies/ associations relevant to ADHD (e.g. American Academy of Pediatrics, American Psychiatric Association, European Psychiatric Association, etc.). Additionally, we will also conduct internet searches for CPGs published online only.

Types of study to be included

Clinical Practice Guidelines

Condition or domain being studied

ADHD in children and adolescents. The inclusion or exclusion of adults will have been at the discretion of the Source CPG developers and age limits, co-morbidities, etc, should have been defined by them. We will assess how this was reported as part of our appraisal using the AGREE II Instrument.

Participants/population

The participants, including inclusion and exclusion criteria, should have been specified by the guideline developers and cannot be known until we begin the appraisal of the guidelines identified by our searches. We will include only CPGs with a section targeting children and adolescents. Relevant items of the AGREE II Instrument will be used to assess how well the scope of the CPG was reported.

Intervention(s), exposure(s)

The interventions included within the included CPGs will have been pre-defined by the CPG development group to match the individual requirements within the scope. We will only consider CPGs that include the comprehensive management of ADHD. The criteria of the AGREE II Instrument will be used to assess how well the included interventions were reported and presented. Identified key target interventions include diagnosis and assessment (i.e. parent, carer, teacher, or patient complaints, history and physical

examination, psychological tools, differential diagnosis, and investigations) and treatment (i.e. psychosocial interventions, pharmacological treatment, comorbidities, parents/ carers and home, monitoring, special cases, transition of care from childhood to adulthood, complementary medicine, and treatment of complications of pharmacological treatment)

Comparator(s)/control

Comparator(s) and control groups with inclusion and exclusion criteria may or may not have been pre-defined by the CPG development group. Comparators would not usually be defined when multiple interventions are included in the recommendations of the CPG.

Primary outcome(s)

(1) Standardize evidence-based assessment and management of ADHD including Early diagnosis and intervention of ADHD and Multi-modal interventions for ADHD.
(2) Improve patient target outcomes and safety (e.g. symptoms of ADHD, academic performance, social relationships, parent-child interactions and family stress, and risk for serious accidental injury)

Secondary outcome(s)

(1) Decrease variation of practice.
(2) Improve patient-related adverse health outcomes that co-occur with ADHD (e.g. substance abuse disorder and smoking, sleep difficulties/disorders, physical injuries, etc.)

Data extraction (selection and coding)

Five reviewers including one CPG methodologist will independently screen the titles and abstracts of all searched documents and determine the ones for full-text review and eligible CPGs for ADHD. Disagreements will be resolved through discussion and consensus with the reviewers. The same five reviewers will search the internet for CPGs published online only. Information relevant to rating of each of the 23 items on the AGREE II Instrument will be extracted from included CPGs by utilizing the online tool (MY AGREE Plus) that is freely available and accessible from the AGREE Enterprise website (http://www.agreetrust.org/). The authors will then independently score every item on a 1-7 scale based on how well each CPG addresses the listed questions and the AGREE criteria. An additional validation of the included CPGs will be conducted for inclusion of systematic reviews in the evidence-based with their category whether Cochrane systematic reviews or others.

Risk of bias (quality) assessment

The AGREE II Instrument addresses the reporting of CPGs. It does not assess aspects of study design quality such as appropriateness of the methods used or trustworthiness of the recommendations. We are not aware of any validated instrument for assessing risk of bias of CPGs. Some of the items on the AGREE II checklist are relevant to the detection of potential sources of bias (e.g. bias due to under-representation of some stakeholders in the development process - items 4 and 5, reporting bias - items 9-12, bias due to conflict of interest/independence of funding bodies - items 22 and 23).

Strategy for data synthesis

Descriptive statistics will be generated and presented in tabular format to summarize the characteristics of the CPGs eligible for inclusion. Once all seven AGREE appraisers have scored each item on the AGREE II, they will hold a meeting to discuss discrepancies between scores and address any obvious discrepancies and errors. For every included CPG, we will use the 'My AGREE PLUS' (http://www.agreetrust.org/my-agree/) to record scores and to keep online records of individual appraiser's scores. For every single CPG, a standardized quality score will be calculated for each of the six domains of the AGREE II Instrument, using the equation presented in the AGREE II User’s Manual. In summary, standardized domain scores will be calculated by summing up all the appraisers’ scores of the individual items in a domain and by scaling the total as a percentage of the maximum possible score for that domain. This calculation is automatically generated on the My AGREE PLUS platform. The overall quality of the included CPGs in each domain of the AGREE II will be presented through the two overall assessments included in the instrument. Since the six domains of the AGREE II are independent, standardized domain quality scores will not be aggregated.

Analysis of subgroups or subsets
The references section of included CPGs will be reviewed and assessed for inclusion of 'Systematic Reviews' in the evidence-base of these CPGs (especially the Cochrane systematic reviews).

Contact details for further information
Dr. Yasser Sami Amer
yasser3amer@yahoo.com

Organisational affiliation of the review
The Saudi ADHD Society, Riyadh, Saudi Arabia
http://adhd.org.sa/en/

Review team members and their organisational affiliations
Dr Turki Albatti. Department of Psychiatry, King Saud University Medical City, King Saud University, Riyadh, Saudi Arabia
Dr Saleh Al Salehi. Child Development Center, King Abdullah Bin Abdulaziz University Hospital, Princess Noura bint Abdulrahman University, Riyadh, Saudi Arabia
Dr Fahad Bashiri. Division of Neurology, Department of Pediatrics, College of Medicine, King Saud University Medical City, King Saud University, Riyadh, Saudi Arabia
Dr Muddathir Hamad. Division of Neurology, Department of Pediatrics, College of Medicine, King Saud University Medical City, King Saud University, Riyadh, Saudi Arabia
Dr Haya Al-Joudi. Department of Neurosciences, King Faisal Specialist Hospital and Research Center, Riyadh, Saudi Arabia
Dr Hadeel Daghash. Ada'a Program, Assistant Deputyship for Hospital Services, Ministry of Health, Riyadh
Mr Jeremy Varnham. Saudi ADHD Society, Riyadh, Saudi Arabia and School of Psychology, University of East London, UK
Dr Yasser Amer. Quality Management Department and Research Chair for Evidence-Based Health Care and Knowledge Translation, King Saud University Medical City, King Saud University, Riyadh, Saudi Arabia - Alexandria Center for Evidence-Based Clinical Practice Guidelines, Faculty of Medicine, Healthcare Quality Directorate, Alexandria University Hospitals, Alexandria University, Alexandria, Egypt

Anticipated or actual start date
04 March 2017

Anticipated completion date
30 November 2017

Funding sources/sponsors
The unified ADHD Clinical Practice Guidelines Project is the strategic project 7.2 of the Saudi ADHD Society for the period 2017-2019. The Saudi ADHD Society is a registered non-profit under licence 474 from the Saudi Ministry of Labor and Social Development, and the project received the Ministry approval (No. 52476) on 5/8/1438.
The project is entirely funded by the Saudi ADHD Society.
No funding received from any pharmaceutical or industrial company

Conflicts of interest
None known

Language
English

Country
Saudi Arabia

Stage of review

Review_Ongoing

Subject index terms status

Subject indexing assigned by CRD

Subject index terms

Attention Deficit Disorder with Hyperactivity; Humans

Date of registration in PROSPERO

24 November 2017

Date of publication of this version

05 October 2017

Details of any existing review of the same topic by the same authors

Stage of review at time of this submission

Stage	Started	Completed
Preliminary searches	Yes	Yes
Piloting of the study selection process	Yes	Yes
Formal screening of search results against eligibility criteria	Yes	Yes
Data extraction	No	No
Risk of bias (quality) assessment	No	No
Data analysis	No	No

Versions

05 October 2017

PROSPERO
This information has been provided by the named contact for this review. CRD has accepted this information in good faith and registered the review in PROSPERO. CRD bears no responsibility or liability for the content of this registration record, any associated files or external websites.
