# Supplementary material for: Appraisal of clinical practice guidelines for the management of attention deficit hyperactivity disorder (ADHD) using the AGREE II Instrument: A systematic review
Source: PLoS One. 2019 Jul 5;14(7):e0219239. doi: 10.1371/journal.pone.0219239 (PMC6611626; doi:10.1371/journal.pone.0219239)
Supplement: S2 Table — (DOCX) [file pone.0219239.s004.docx]

| **S2 Table. Mapping of ADHD CPGs against their citation of systematic reviews, meta-analyses and their utilization of the GRADE method during development** | | | |
| --- | --- | --- | --- |
| **CPG was developed using the GRADE method** | **Number of Cochrane reviews (% of total systematic review)** | **Number of systematic reviews with or without meta-analysis cited in the references section** | **ADHD CPG Developer Organization** *(date of the last update)* |
| ***No*** | ***Nil (0)*** | ***TWO***  5 (Charach 2011), 45 (Cheng 2007) | **AAP** *2011* |
| ***No*** | ***Nil (0)*** | ***SEVEN***  11 (Polanczyk 2007), 239 (Cortese 2006) 38 (Connor 2002), 151 (Gaub 1997), 206 (Jerome 2006), 211 (Biederman 2007), 248 (Consoli 2007) | **CADDRA**  **Third edition**  *2010* |
| ***No***  But starting from 2016 NHMRC started developing their CPGs using the GRADE  <https://www.nhmrc.gov.au/guidelines-publications/how-nhmrc-develops-its-guidelines> | ***Five (36%)*** | ***FOURTEEN***  **104 (Li 2011 Coch)**  **103 (Krisanaprakornkit 2010 Coch)**  102 (Davidson 2011)  **101 (Heirs 2007 Coch)**  99 (Karpouzis 2010)  98 (Raz 2009)  58 (King 2006 HTA)  **53 (Storebo 2011 Coch)**  52 (Cartwright-Hatton 2004)  **51 (Bjornstad 2005 Coch),**  48 (Fabiano 2009)  54 (Klein 2007)  59 (Faraone 2009)  60 (Schachter 2001) | **NHMRC** *2012* |
| ***Yes***  +SIGN criteria for some recommendations | 19 (28%) | See separate file for the 2018 updated CPG for details  67 | **NICE**  *(last update 16th March 2018)* |
| **No** | ***Four*** (24%)  74, 98, 142, 179 | ***SEVENTEEN***  58 (Montoya 2011), **74 (Zwi 2011 Coch),** **98 (Storebo 2011 Coch),** 111 (Bloch 2011), 116 (Ghanizadeh 2013), 126 (Willis 2011), 136 (Ghanizadeh 2011), 140 (Lee 2011), **142 (Li 2011 Coch),** 171 (Westover 2012), **179 (Pringsheim 2011 Coch**), 181 (Wilens 2008), 184 (Reichow 2013), 205 (Pringsheim 2012), 223 (Treuer 2013), 237 (Wu 2012), 238 (King 2006), | **Singapore MOH**  *August 2014* |
| ***No*** | ***0***  Although two Cochrane systematic reviews were mentioned in the text of the CPG, they were not cited in the references section | ***ONE***  Pliszka 2007 | **UMHS**  *April 2013* |

- Total number of ADHD-related systematic review protocols registered in the ***PROSPERO database*** is (417: any review status), using the SEARCH function by PROSPERO as of 26/6/2019, including the following:-
- Ongoing (337)
- Completed but not published (35) including this study
- Completed and published (39)
- Discontinued (2)
- Ongoing update (1)

**NICE CPG**

**2018 evidence review**

<https://www.nice.org.uk/guidance/ng87/evidence/march-2018-evidence-reviews-172212706836?tab=evidence>

|  | **Systematic review/ Meta-analysis** | | **Cochrane** | |
| --- | --- | --- | --- | --- |
|  | Nil | 0 | Nil | **0** |
|  | 1. 23 Butler 2015 2. 36 Corcoran 2016 3. 68 Gwernan-Jones 2016 4. 109 Laugesen 2016 5. 154 Richardson 2015 6. 159 Schatz 2015 | 6 | Nil | **0** |
|  | 1. 66 Barnard 2002 2. 94 Beiderman 2007 3. **144 Castells 2011 COCH** 4. **159 Ching 2012 COCH** 5. 166 Coghill 2010 6. 205 Dinca 2005 7. 226 Faraone 2009 8. 227 Faraone 2010 9. 256 Fung 2016 10. **338 Hurwitz 2012 COCH** 11. 372 King 2006 HTA 12. 464 Moriyama 2013 13. 492 Parker 2013 14. 499 Peterson 2008 15. **512 Pringsheim 2011 COCH** 16. **513 Punja 2012 COCH** 17. **520 Redman 2014 COCH** 18. 521 Reichow 2013 19. **627 Thomson 2009 COCH** 20. **628 Thomson 2009 COCH** 21. 642 Van der Oord 2008 22. 715 Zimovetz 2012 | 22 | 1. **144 Castells 2011 COCH** 2. **159 Ching 2012 COCH** 3. **338 Hurwitz 2012 COCH** 4. **512 Pringsheim 2011 COCH** 5. **513 Punja 2012 COCH** 6. **520 Redman 2014 COCH** 7. **627 Thomson 2009 COCH** 8. **628 Thomson 2009 COCH** | **8** |
|  | 1. **164 Ching 2012 COCH** 2. 173 Coghill 2010 3. 212 Dinca 2005 4. 262 Fung 2016 5. **346 Hurwitz 2012 COCH** 6. 470 Moriyama 2013 7. 496 Parker 2013 8. **517 Pringsheim 2011 COCH** 9. **518 Punja 2012 COCH** 10. **525 Redman 2014 COCH** 11. 526 Reichow 2013 12. **629 Thomson 2009 COCH** 13. **630 Thomson 2009 COCH** | 13 | 1. **164 Ching 2012 COCH** 2. **346 Hurwitz 2012 COCH** 3. **517 Pringsheim 2011 COCH** 4. **518 Punja 2012 COCH** 5. **525 Redman 2014 COCH** 6. **629 Thomson 2009 COCH** 7. **630 Thomson 2009 COCH** | **7** |
|  | 1. **78 Butler 2015** 2. **86 Cerrillo-Urbina 2015** 3. **105 Coates 2015** 4. **117 Daley 2014** 5. **198 Gwernan-Jones 2016** 6. **274 Krisanaprakornkit 2010 COCH** 7. **282 Laugesen 2016** 8. **294 Li 2011 COCH** 9. **353 Parker 2013** 10. **394 Schatz 2015** 11. **430 Sonuga-Barke 2013** 12. **444 Storebǿ 2011 COCH** 13. **453 Tatlow-Golden 2016** 14. **467 Van der Oord 2008** 15. **475 Vollebregt 2014** 16. **506 Zwi 2009 COCH** | 16 | 1. **274 Krisanaprakornkit 2010 COCH** 2. **294 Li 2011 COCH** 3. **444 Storebǿ 2011 COCH** 4. **506 Zwi 2009 COCH** | **4** |
|  | 1. **31 King 2006** | 1 | Nil | **0** |
|  | 1. **23 Butler 2015** 2. **36 Corcoran 2016** 3. **68 Gwernan-Jones 2016** 4. **108 Laugesen 2016** 5. **156 Schatz 2015** | 5 | Nil | **0** |
|  | 1. **23 Butler 2015** 2. **66 Gwernan-Jones 2016** 3. **107 Laugesen 2016** 4. **154 Schatz 2015** | 4 | Nil | **0** |
|  | Nil | 0 | Nil | **0** |
| **TOTAL** | | **67** |  | **19 (28%)** |
