# Supplementary material for: Appraisal of clinical practice guidelines for the management of attention deficit hyperactivity disorder (ADHD) using the AGREE II Instrument: A systematic review
Source: PLoS One. 2019 Jul 5;14(7):e0219239. doi: 10.1371/journal.pone.0219239 (PMC6611626; doi:10.1371/journal.pone.0219239)
Supplement: S3 Table — (DOCX) [file pone.0219239.s005.docx]

**S3 Table. Key recommendations of included ADHD CPGs**

| **CPGs** | **Diagnosis** | **Treatment** |
| --- | --- | --- |
| **AAP**  (37) | - The primary care clinician should initiate an evaluation for ADHD for any child 4 through 18 years of age who presents with academic or behavioral problems and symptoms of inattention, hyperactivity, or impulsivity (quality of evidence B/strong recommendation*). - To make a diagnosis of ADHD, the primary care clinician should determine that Diagnostic and Statistical Manual of Mental Disorders, Fourth Edition criteria have been met (including documentation of impairment in more than 1 major setting); information should be obtained primarily from reports from parents or guardians, teachers, and other school and mental health clinicians involved in the child’s care. The primary care clinician should also rule out any alternative cause (quality of evidence B/strong recommendation). - In the evaluation of a child for ADHD, the primary care clinician should include assessment for other conditions that might coexist with ADHD, including emotional or behavioral (e.g., anxiety, depressive, oppositional defiant, and conduct disorders), developmental (e.g., learning and language disorders or other neurodevelopmental disorders), and physical (e.g., tics, sleep apnea) conditions (quality of evidence B/strong recommendation). - The primary care clinician should recognize ADHD as a chronic condition and, therefore, consider children and adolescents with ADHD as children and youth with special health care needs. Management of children and youth with special health care needs should follow the principles of the chronic care model and the medical home (quality of evidence B/strong recommendation).   **For the classification of the levels of evidence and grades of recommendations, refer to the original CPG document.* | **Recommendations for treatment of children and youth with ADHD vary depending on the patient’s age:**   - - **For preschool-aged children (4–5 years of age):** the primary care clinician should prescribe evidence-based parent- and/or teacher-administered behaviour therapy as the first line of treatment (quality of evidence A/strong recommendation) and may prescribe MPH if the behavior interventions do not provide significant improvement and there is moderate-to-severe continuing disturbance in the child’s function. In areas where evidence-based behavioral treatments are not available, the clinician needs to weigh the risks of starting medication at an early age against the harm of delaying diagnosis and treatment (quality of evidence B/recommendation).   - **For elementary school–aged children (6–11 years of age):** the primary care clinician should prescribe US Food and Drug Administration–approved medications for ADHD (quality of evidence A/strong recommendation) and/or evidence-based parent- and/or teacher-administered behavior therapy as treatment for ADHD, preferably both (quality of evidence B/strong recommendation). The evidence is particularly strong for stimulant medications and sufficient but less strong for ATX, extended-release GUA, and extended-release clonidine (in that order) (quality of evidence A/strong recommendation). The school environment, program, or placement is a part of any treatment plan.   - **For adolescents (12–18 years of age):** the primary care clinician should prescribe Food and Drug Administration–approved medications for ADHD with the assent of the adolescent (quality of evidence A/strong recommendation) and may prescribe behavior therapy as treatment for ADHD (quality of evidence C/recommendation), preferably both. - The primary care clinician should titrate doses of medication for ADHD to achieve maximum benefit with minimum adverse effects (quality of evidence B/strong recommendation). |
| **UMHS** (38) | **Types:**  Diagnosis is based on the DSM-V criteria [D*]. The three main types are primary hyperactive, primary inattentive, and combined.  **Multiple sources:**  No specific test can make the diagnosis. Input from both parents and teachers or other source is required. Some psychological rating tools are useful but are not diagnostic (e.g., Vanderbilt, Conners). If a learning problem is suspected, consider neuropsychiatric testing for intelligence testing (IQ) and learning disorders.  **Confused and associated conditions:**  Diagnosis is complicated by overlapping symptoms or co-occurrence of other disorders (e.g., anxiety disorders, bipolar disorder, obstructive sleep apnea, fetal alcohol syndrome, major depressive disorders, learning disorders, oppositional defiant disorder, post-traumatic stress disorder, reactive attachment disorder).  **For the classification of the levels of evidence and grades of recommendations, refer to the original CPG document.* | **Drug treatment**   - Stimulants are the first line treatment and have proven benefit to most people. If one class of stimulant fails or has unacceptable side effects then another should be tried [IA*]. - ATX is a secondary choice [IA]. (One reported side effect is suicidal thinking.) - Other medications may be used alone or in combination depending upon the ADHD type, response to therapy or comorbidity profile: e.g., Alpha-II agonists (clonidine, GUA) with hyperactivity or impulsivity; bupropion (over age 8) with co-morbid depression; risperidone (atypical antipsychotic) for aggression [IIA]. - Comorbid conditions may require additional treatment (e.g., for depression) and consideration of referral to a mental health specialist.   **Non-pharmacologic interventions:**   - **Age-appropriate behavioral interventions at home:** education and support [IB]; parent interventions including routines, clear limits and positive reinforcement for target behaviors (for children); consider family therapy; cognitive behavioral techniques for adults [IIB]. - **School interventions:** children with ADHD may qualify for a 504 education plan or special education services with individualized education plan (IEP)[ID].   **Special Populations or Circumstances:**  Special considerations apply to: 3-5 year olds, adolescents and adults, head-injured, intellectually disabled/autistic, fetal alcohol syndrome, and substance-abusing patients.  **Controversial Areas:**  **Common myths.** Several common beliefs related to ADHD are untrue, e.g., that it is not a real disorder, it is an over-diagnosed disorder, children with ADHD are over-medicated.  **Diets.** Although a few studies suggest dietary modification may have promise, there is no proof of efficacy (e.g., individually tailored hypoallergenic diets, essential fatty acids, flax seed) [IIB*], studies have shown the Feingold diet and modifying sugar consumption have no effect [IIIB].  **Complementary Alternative Medicine.** Use is controversial, but common. |
| **CADDRA** (39) | **Diagnosis of ADHD***   - ADHD patients can be managed in a primary care setting according to Diagnostic and Statistical Manual 5 (DSM-5) Criteria of ADHD Symptoms. - Overall psychiatric health should always be considered and a risk assessment done at the onset. - Three DSM-5 presentations exist: inattentive presentation, hyperactive-impulsive presentation, and combined presentation. - Red flags of ADHD should be identified and addressed - Steps include: initial information gathering, medical review, ADHD-specific interview, feedback and treatment recommendations   **Differential diagnosis and comorbid disorders**   - Twelve comorbid conditions have been identified according to the age group. - Comorbid conditions that have overlapping symptoms with ADHD are common   **Special considerations across the lifespan**   - Impact/functional disability across the lifespan - Accidents/risks - Driving   **For further details of the recommendations, refer to the original CPG document and toolkit in the official website.* | **Psychosocial treatment of ADHD**   - CADDRA guide to ADHD Psychoeducation includes key elements: Discover, Demystify (Myths And Facts), Instill Hope, Educate, Empathize, Encourage, Guide and Motivate, Be culturally and gender sensitive, Promote a balanced lifestyle, and lastly, Give Online Resources / Local Community Resources / Book Lists. - Psychosocial interventions at home (instructional, behavioral, and environmental interventions), school (instructional, behavioral, environmental, academic, executive function, post-secondary interventions), workplace (workplace interventions). - Manualized interventions include Parent Management Training Models; Social Skills Training; Cognitive Behavioral Therapy; and Mindfulness Training.   **Pharmacological treatment of ADHD**  **Medication classification**   - **First-Line Treatments:** Long-acting psychostimulants (MPH and amphetamines). CADDRA therefore recommends an adequate trial of both classes of long-acting psychostimulants before engaging in a trial of a second-line treatment. - **Second-Line Treatments**: ATX, Guanfacine XR and short/intermediate acting psychostimulants. They can be used for patients who experience significant side effects, have had suboptimal response with first-time medications, or do not have access to first-line medications. Non-stimulants may also be used in combination with first-line agents as a potential augmentation for first-line treatment suboptimal responders. Second-line non-stimulant agents also are appropriate where stimulant agents are contraindicated, such as in cases where there is high risk of stimulant misuse - **Third-Line Treatments:** Bupropion, clonidine, imipramine and modafinil are examples. Atypical antipsychotics are among agents used for comorbidities commonly seen with ADHD, often in combination with other agents. - Stepped approach to prescribing includes: Setting Treatment Objectives; Medication Selection; Titration & Monitoring; and Ongoing Follow-up.   *For details on the sections for Managing side effects, Common Adverse Events, When to Reduce the Dose, or Stop a Medication, How to Stop Medication, Choosing to Change to a Different Medication, Side Effects Management Techniques, unsatisfactory response to treatment, information on specific medications, and treatments requiring further research, refer to the full CPG document from CADDRA website.* |
| **NHMRC** (40) | **Diagnosis:**   - The DSM-IV or ICD10 criteria must be met for a diagnosis of ADHD to occur. - This requires that the symptoms of hyperactive, impulsive and inattentive behaviour must be deemed by a specialist clinician to:   - Have had their onset in early childhood (before age of 7)   - Be maladaptive and excessive for the child/adolescent’s age and developmental level   - Have persisted over time (at least 6 months)   - Be evident in more than one setting   - Have caused significant functional impairment   - Have no better alternative explanation, such as another mental disorder. - The process leading to diagnosis of ADHD should also include a physical examination, and a holistic assessment of the individual’s needs, family, social and educational circumstances and coexisting conditions. - Information should be gathered from multiple sources, particularly the child/adolescent’s school. - Diagnosis in young children (under 7 years) should be made by a specialist clinician who observes the child over several months and reviews them as they start school.   **Assessment and case formulation:**   - ADHD is a description rather than an explanation of a pervasive, persistent, disabling pattern of inattentiveness, over-activity and/or impulsivity. A child/adolescent who meets diagnostic criteria for ADHD may not be always best served by making that diagnosis. For example, their behaviour could be understood as a reaction to specific cognitive difficulties or family/environmental circumstances. - Assessment requires establishing evidence of impairment across multiple settings, via gathering information from multiple informants including the child/adolescent, health professionals, parents/ carers and teachers. - GPs may carry out the initial assessment. If, on the basis of this assessment it is suspected that a child/adolescent has ADHD and/or has behavioural, emotional or cognitive symptoms causing significant and persistent impairment to them, the family or at school, the GP can provide information and referral to a specialist clinician and parent training/education or support programmes for the family (this can precede a formal diagnosis of ADHD). - Thorough assessment for possible ADHD requires a specialist clinician to consider:   - A comprehensive medical, developmental and mental health assessment of the child/adolescent   - A psychosocial assessment of the child/adolescent and their family   - a cultural and social assessment of the meaning and significance of the behaviours   - Seeking out available explanations for the presentation, including but not restricted to alternative medical diagnoses   - Whether the symptoms are developmentally excessive for the child’s developmental age and associated expectations   - Any comorbid diagnosis. - If indicated (based on the assessments above), additional assessment may be required. In this circumstance, the specialist clinician should consider:   - A cognitive and behavioural assessment   - Allied health and educational assessments   - The assistance of a cultural interpreter or Aboriginal and Torres Strait Islander health worker. | *For the ‘general principles of management’ refer to the full CPG document from NHMRC.*  **Psychological management:**   - Where indicated, young children (under 7 years) and school age children diagnosed with ADHD, and their families, may benefit from evidence-based psychological interventions that have demonstrated effectiveness for associated mental health problems. Such interventions can improve outcomes for internalising (emotional) and externalising (behavioural) symptoms. Not all psychological approaches have an evidence base, and only those where this is the case should be implemented. - In prioritising intervention and management, consideration should be given to the ability of the child/adolescent and their parents/carers to implement strategies. These interventions may be impractical for families due to time demands and cost associated with them or may not be locally available particularly in some rural areas. - In adolescents diagnosed with ADHD cognitive behavioural therapy may improve internalising symptoms. - The acceptability and the effectiveness of these interventions should be monitored including following up the child after the psychological treatment has finished and providing relapse prevention booster sessions if indicated.   **Pharmacological management:**   - Use of stimulant medications (MPH and dexamphetamine sulphate) can reduce core ADHD symptoms and improve social skills and peer relations in children and adolescents diagnosed with ADHD in the short term (up to 3 years). - Not all children and adolescents with ADHD will require, or benefit from, pharmacological management. The use of clinical judgement is required to evaluate the harms versus benefits of stimulant use for each individual case upon discussion with the child/adolescent and their family. - If medication is to be used in management, stimulants are presently the first line of treatment. - If pharmacological treatment is implemented, it should be based on a comprehensive assessment under the direction of a paediatrician (including a paediatric neurologist), or child/adolescent psychiatrist. - Before prescribing stimulants to a child/adolescent diagnosed with ADHD, the clinician should consider:   - Baseline physical assessment data, including, as a minimum, pulse, blood pressure, weight and height. Change in weight and height should be followed over time using centile charts. If there are any abnormal symptoms, findings or history regarding cardiovascular status, appropriate investigation and referral should be organised   - The specific needs and expressed preferences of the child/adolescent, and the circumstances of their family and culture   - Underlying or associated psychosocial problems, educational difficulties, cognitive profile and/or comorbid conditions   - Potential short-term benefits   - Likelihood of compliance   - Potential harms, allergies, adverse effects and contraindications, including diversion of medications for misuse and abuse   - Duration of treatment and signals to stop treatment   - Schedule for follow-up, monitoring and review. - These factors, as well as the risks and benefits of medication and what to do if they have concerns about treatment, need to be directly discussed with the child/adolescent and their parents/carers. Following this discussion, if drug treatment is not accepted by the child/adolescent or their parent/carers, alternatives should be discussed if this has not already been done. - If the maximum dose (after titration) has been reached and feedback from parents/carers (and if possible teachers), suggest that there is no significant improvement after a month of treatment, then alternative treatments should be considered. - The optimal dose of stimulant medication can be titrated against clinical benefit and may be prescribed in various forms of the medications, including: immediate release, extended release or both. - When stimulant treatment is used it should only be continued if there is demonstrated benefit in the absence of unacceptable side effects. - If medication is stopped, regular assessments are needed to assess the child/adolescent’s responses across multiple settings. - Children/adolescents on stimulant medication require 3-6 monthly clinical assessment and review to ensure the management strategies remain appropriate and effective. Monitoring should include assessment of side effects and particularly psychological symptoms and plotting of growth parameters, pubertal development, heart rate and blood pressure. - If it is indicated that a child/adolescent no longer requires stimulant medication, then the clinician should discuss trialling such a period off medication with the child/adolescent and their parents/carers and teachers. This trial should last at least several weeks and begin at an appropriate time. - Consideration of the changing needs of patients with ADHD as they transition through school and into adult life is imperative to the provision of optimal clinical care. - For young children (under 7 years) psychological, environmental and family interventions should, if possible, be trialed and evaluated before initiating pharmacological treatment. If all these other interventions have not been effective then stimulants might be considered for this age group in consultation with the parents or guardians and including when appropriate teachers or other carers. - If medication is prescribed for young children (under 7 years), it should be started with a low dose and regularly monitored; for example, initially weekly by the GP in consultation with the specialist to assess improvements and the presentation of any adverse effects, then every 2-3 months if benefits from medication have been demonstrated.   **Educational management:**   - In terms of improving behavioural and academic outcomes, children/adolescents may benefit from individually tailored modifications to the educational setting and curriculum, as informed by the overall case formulation. These modifications could include classroom based approaches that restructure how information is presented to students with ADHD and should form part of an individual education plan for the child (or its equivalent), at their school. |
| **SMOH** (41) | **Definition and diagnostic classification:**   - [B*] A diagnosis of ADHD should be made through a thorough clinical assessment, which should include an interview with the parent or significant caregiver of the child. Grade B, Level 2++ - [B] When diagnosing attention deficit hyperactivity disorder, in addition to information from interviews with parents or caregivers, information from another adult who has interacted with the child in another setting (e.g. school teachers) should also be obtained. Grade B, Level 2++ - [B] Before diagnosing ADHD, a careful evaluation to exclude psychiatric or medical conditions which can account for ADHD-like symptoms should be performed. Grade B, Level 2++ - [B] ADHD is a diagnosis that should be considered when a pre-schooler presents with disruptive behaviour. Grade B, Level 2++ - [C] The clinician should assess a child diagnosed with ADHD for co-morbid conditions. Grade C, Level 2+ - [C] If there is a suspected learning disorder, appropriate psycho-educational or speech and language assessments should be sought from the appropriate specialists. Grade C, Level 2+ - [A] There is no need for investigations such as thyroid function test, lead level or brain imaging to be done when assessing a child for attention deficit hyperactivity disorder, unless there is another medical indication. Grade A, Level 1+ - [A] Electroencephalogram is not recommended as a diagnostic tool for ADHD in clinical practice. Grade A, Level 1+   **For the classification of the levels of evidence and grades of recommendations, refer to the original CPG document.* | **Overview of treatment for ADHD:**   - [D] Clinicians who treat adolescents with ADHD should plan for the transition to adult health services in advance, discuss this with the patients and their families, and ensure that they can continue to receive care. Grade D, Level 4   **Psychosocial/alternative/complementary interventions:**   - [B] After diagnosis, doctors should provide appropriate education about ADHD to children, families and teachers. Grade B, Level 1+ - [A] Doctors should consider educating parents of children with ADHD about behaviour management strategies, or refer them to professionals who can do so [e.g. psychologists] Grade A, Level 1++ - [B] Parent training should be offered for parents of pre-school children with attention deficit hyperactivity disorder. Grade B, Level 1+ - [B] Doctors should consider referring parents of children and adolescents with ADHD for parent training programmes offered within the community, particularly when negative parenting practices are identified. Grade B, Level 1+ - [D] During the delivery of parent training, the professional should consider the use of behaviour management strategies which are more likely to be acceptable to the parents, based on an understanding of their cultural background. Grade D, Level 3 - [GPP] Family therapy may be considered for the family of a child or adolescent with ADHD if severe disruption in relationships within the family is evident. GPP - [C] Academic interventions should be considered for the child with ADHD and should be made in consultation with educational professionals who work closely with the child in the learning or school context. Grade C, Level 2+ - [GPP] Parents and caregivers should be encouraged to actively share information about the child’s ADHD condition with his school, and collaborate with professionals and teachers in preparing the child for the educational setting. GPP - [B] Social skills training alone is not recommended for the management of attention deficit hyperactivity disorder. Grade B, Level 1+ - [B] Cognitive-behavioural therapy alone is not recommended for the management of attention deficit hyperactivity disorder. Grade B, Level 1+ - [B] There is no clear evidence for food additives and sugars to be related to attention deficit hyperactivity disorder. Parents and children should be advised to control food items containing additives or high sugar content that have been observed to consistently provoke physical or behavioural reactions. Grade B, Level 1+ - [C] A restrictive elimination diet is not recommended for the management of attention deficit hyperactivity disorder. Grade C, Level 2++ - [B] Omega-3 supplementation may be used as an adjunctive treatment for attention deficit hyperactivity disorder. Grade B, Level 1+ - [B] Neurofeedback should not be used alone for the treatment of attention deficit hyperactivity disorder. Grade B, Level 1+ - [A] Cognitive remediation alone is not recommended for the treatment of ADHD with significant impairment. Grade A, Level 1+ - [D] A referral to an Occupational Therapist may be considered for children with sensory processing or motor skill deficits in addition to attention deficit hyperactivity disorder. Grade D, Level 3   **Pharmacological treatment:**   - [A] When medication is considered for the treatment of attention deficit hyperactivity disorder, MPH should be considered first. Grade A, Level 1+ - [B] MPH may be used for long term treatment of ADHD symptoms, although the benefits of treatment should be reviewed regularly. Grade B, Level 1+ - [B] Drug holidays during treatment with MPH may be considered in order to limit adverse effects. ADHD symptoms and impairment during the non-medication days should be monitored. Grade B, Level 1+ - [A] The height, weight and body-mass-index (BMI) of children receiving treatment with MPH should be regularly monitored. Grade A, Level 1++ - [D] The height, weight and body-mass-index (BMI) of children receiving treatment with MPH should be monitored every 6 months. If there is concern about slowing of growth rate, the need for continued medication use should be reviewed and jointly decided with parents, and there may be a need to evaluate for other medical reasons explaining this. Grade D, Level 4 - [B] During treatment with MPH, start at a low dose and slowly titrate upwards according to the child’s response, or adjust the timing of medication, to minimise short-term adverse effects. Grade B, Level 1+ - [C] A careful personal and family history of cardiovascular disease should be taken before starting medication treatment for attention deficit hyperactivity disorder. Children with pre-existing cardiac problems should be referred to a cardiologist for evaluation before treatment with MPH or ATX is initiated. Grade C, Level 2+ - [A] MPH may be used to treat ADHD in children with comorbid tic disorder but treatment should be stopped if the tics worsen following treatment. Grade A, Level 1+ - [B] The use of MPH should be considered when treating ADHD in the presence of co-morbid disruptive behavioural disorder. Grade B, Level 1+ - [B] The use of an extended-release MPH instead of immediate- release MPH should be considered if there is concern about medication abuse. Medication use by these patients should be carefully monitored. Grade B, Level 1+ - [A] MPH may be considered for the treatment of ADHD in individuals who have also been diagnosed with autistic spectrum disorder. Care should be taken to watch for side effects. Grade A, Level 1+ - [A] ATX may be used for the treatment of ADHD symptoms when there is increased risk with MPH use [e.g. high risk of abuse or diversion]. Grade A, Level 1+ - [A] During treatment with ATX, there should be periodic monitoring of growth (height and weight) and mental state (suicidal thinking). If there is concern about slowing of growth rate, the need for continued medication use should be reviewed and jointly decided with parents, and there may be a need to evaluate for other medical reasons explaining this. Grade A, Level 1++ - [D] The height, weight and body-mass-index (BMI) of children receiving treatment with ATX should be monitored every 6 months. Grade D, Level 4 - [B] ATX may be used as first line treatment when there is comorbid ADHD and tic disorder. Grade B, Level 1+ - [C] The combination of MPH and ATX should not be used for the treatment of ADHD symptoms. Grade C, Level 2+ - [A] To improve treatment adherence, treatment should be individualised for each patient with attention deficit hyperactivity disorder, and the parents’ and their child’s preferences should be considered. Grade A, Level 1+ - [A] The use of MPH or ATX in pre-schoolers should be considered only if psychosocial interventions have failed. Care should be taken to regularly assess response and monitor for side effects, so as to decide if medication should continue to be administered. Grade A, Level 1++   **Cost-effectiveness issues**   - [A] Although medication is a cost-effective treatment for ADHD, treatment for ADHD should be individualised and other factors (e.g. presence of co-morbidity) should be considered before initiating medications. Grade A, Level 1++ |
| **NICE** (42) | **Identification and referral:**   - Universal screening for ADHD should not be undertaken in nursery, primary and secondary schools. - When a child or young person with disordered conduct and suspected ADHD is referred to a school's special educational needs coordinator (SENCO), the SENCO, in addition to helping the child with their behaviour, should inform then parents about local parent-training/education programmes. - Referral from the community to secondary care may involve health, education and social care professionals (for example, GPs, paediatricians, educational psychologists, SENCOs, social workers) and care pathways can vary locally. The person making the referral to secondary care should inform the child or young person's GP. - When a child or young person presents in primary care with behavioural and/or attention problems suggestive of ADHD, primary care practitioners should determine the severity of the problems, how these affect the child or young person and the parents or carers, and the extent to which they pervade different domains and settings. - If the child or young person's behavioural and/or attention problems suggestive of ADHD are having an adverse impact on their development or family life, consider:-   - A period of watchful waiting of up to 10 weeks   - Offering parents or carers a referral to group-based ADHD-focused support (this should not wait for a formal diagnosis of ADHD).   - If the behavioural and/or attention problems persist with at least moderate impairment, the child or young person should be referred to secondary care (that is, a child psychiatrist, paediatrician, or specialist ADHD CAMHS) for assessment. - If the child or young person's behavioural and/or attention problems are associated with severe impairment, referral should be made directly to secondary care (that is, a child psychiatrist, paediatrician, or specialist ADHD CAMHS) for assessment. - Primary care practitioners should not make the initial diagnosis or start medication in children or young people with suspected ADHD. - Adults presenting with symptoms of ADHD in primary care or general adult psychiatric services, who do not have a childhood diagnosis of ADHD, should be referred for assessment by a mental health specialist trained in the diagnosis and treatment of ADHD, where there is evidence of typical manifestations of ADHD (hyperactivity/impulsivity and/or inattention) that:-   - Began during childhood and have persisted throughout life   - Are not explained by other psychiatric diagnoses (although there may be other coexisting psychiatric conditions)   - Have resulted in or are associated with moderate or severe psychological, social and/or educational or occupational impairment. - Adults who have previously been treated for ADHD as children or young people and present with symptoms suggestive of continuing ADHD should be referred to general adult psychiatric services for assessment. The symptoms should be associated with at least moderate or severe psychological and/or social or educational or occupational impairment.   **Diagnosis:**   - A diagnosis of ADHD should only be made by a specialist psychiatrist, paediatrician or other appropriately qualified healthcare professional with training and expertise in the diagnosis of ADHD, on the basis of:-   - A full clinical and psychosocial assessment of the person; this should include discussion about behaviour and symptoms in the different domains and settings of the person's everyday life and   - A full developmental and psychiatric history and   - Observer reports and assessment of the person's mental state. - A diagnosis of ADHD should not be made solely on the basis of rating scale or observational data. However, rating scales such as the Conners' rating scales and the Strengths and Difficulties Questionnaire are valuable adjuncts, and observations (for example, at school) are useful when there is doubt about symptoms. - For a diagnosis of ADHD, symptoms of hyperactivity/impulsivity and/or inattention should:-   - Meet the diagnostic criteria in DSM-5 or ICD-10 (hyperkinetic disorder) and   - Cause at least moderate psychological, social and/or educational or occupational impairment based on interview and/or direct observation in multiple settings and   - Be pervasive, occurring in 2 or more important settings including social, familial, educational and/or occupational settings.   - As part of the diagnostic process, include an assessment of the person's needs, coexisting conditions, social, familial and educational or occupational circumstances and physical health. For children and young people, there should also be an assessment of their parents' or carers' mental health. - ADHD should be considered in all age groups, with symptom criteria adjusted for age-appropriate changes in behaviour. - In determining the clinical significance of impairment resulting from the symptoms of ADHD in children and young people, their views should be taken into account wherever possible.   *Additional information and support for people with ADHD and their families and carers were provided in the full NICE guidance including sections on involving schools, colleges, universities, and other healthcare professionals.* | **Treatment and management:**   - These recommendations are for health care professionals with training and expertise in diagnosing and managing ADHD.   **Children ˂5 years:**   - **First-line:** offer an ADHD-focused group parent-training programme to parents/carers. - If after an ADHD-focused group parent-training programme, ADHD symptoms across settings are still causing a significant impairment after environmental modifications have been implemented and reviewed, obtain advice from a specialist ADHD service with expertise in managing ADHD in young children, ideally a tertiary service. - **Do NOT** offer medication for ADHD for any child ˂5 years without a second specialist opinion from an ADHD service with expertise in managing ADHD in young children, ideally a tertiary service.   **Children/young people aged ≥5 years:**   - **First-line:** give ADHD-focused information and offer additional group based and ADHD-focused support to parents/carers.   This may be as few as 1 or 2 sessions and should include:   - - Education and information on the causes and impact of ADHD,   - Advice on parenting strategies,   - Both parents and carers if feasible. - If a child/young person has symptoms of oppositional defiant disorder or conduct disorder, offer parents/carers a parent training programme in line with NICE's recommendations on antisocial behaviour and conduct disorders in children/young people as well as group-based ADHD-focused support. - Consider individual parent-training/education programmes for   parents/carers when:   - - There are particular difficulties for families in attending group sessions,   - A family's needs are too complex to be met by group-based parent-training/education programmes. - Offer medication for children/young people aged ≥5 years only if their symptoms are still causing a persistent significant impairment in at least one domain after their parents/carers have received ADHD-focused information, group-based support has been offered and environmental modifications have been implemented and reviewed. - Consider a course of CBT for young people who have benefited from medication but whose symptoms are still causing a significant impairment in at least one domain, addressing the following areas: - Social skills with peers, - Problem-solving, - Self-control, - Active listening skills, - Dealing with and expressing feelings   **Adults:**  These recommendations are for healthcare professionals with training and expertise in diagnosing and managing ADHD.   - Offer medication to adults with ADHD if their ADHD symptoms are still causing a significant impairment in at least one domain after environmental modifications have been implemented and reviewed. - Consider non-pharmacological treatment for adults with ADHD who have: - Made an informed choice not to have medication - Difficulty adhering to medication - Found medication to be ineffective or cannot tolerate it. - Consider non-pharmacological treatment in combination with medication for adults with ADHD who have benefited from medication but whose symptoms are still causing a significant impairment in at least one domain. - When non-pharmacological treatment is indicated for adults with ADHD, offer the following as a minimum: - A structured supportive psychological intervention focused on ADHD - Regular follow-up either in person or by phone.   Treatment may involve elements of or a full course of CBT.  **Dietary advice:**   - Healthcare professionals should stress the value of a balanced diet, good nutrition and regular exercise for children, young people and adults with ADHD. - Do NOT advise elimination of artificial colouring and additives from the diet as a generally applicable treatment for children and young people with ADHD. - Ask about foods or drinks that appear to influence hyperactive behaviour as part of the clinical assessment of ADHD in children and young people, and: - If there is a clear link, advise parents or carers to keep a diary of food and drinks taken and ADHD behaviour. - If the diary supports a relationship between specific foods and drinks and behaviour, offer referral to a dietitian. - Ensure that further management (for example, specific dietary elimination) is jointly undertaken by the dietitian, mental health specialist or paediatrician, and the parent or carer and child or young person. - Do NOT advise or offer dietary fatty acid supplementation for treating ADHD in children and young people. - Advise the family members or carers of children with ADHD that there is no evidence about the long-term effectiveness or potential harms of a 'few food' diet for children with ADHD, and only limited evidence of short-term benefits.   **Pharmacological management:**  **Medication:**  These recommendations, except regarding the ‘risk of stimulant misuse or diversion’, are for healthcare professionals with training and expertise in diagnosing and managing ADHD.   - All medication for ADHD should only be initiated by a healthcare professional with training and expertise in diagnosing and managing ADHD. - Healthcare professionals initiating medication for ADHD should: - Be familiar with the pharmacokinetic profiles of all the short- and long-acting preparations available for ADHD - Ensure that treatment is tailored effectively to the individual needs of the child, young person or adult - Take account of variations in bioavailability or pharmacokinetic profiles of different preparations to avoid reduced effect or excessive adverse effects.   **Baseline assessment:**   - Before starting medication for ADHD, people with ADHD should have a full assessment, which should include: - A review to confirm they continue to meet the criteria for ADHD and need treatment. - A review of mental health and social circumstances, including: - Presence of coexisting mental health and neurodevelopmental conditions - Current educational or employment circumstances - Risk assessment for substance misuse and drug diversion - Care needs - A review of physical health, including: - A medical history, taking into account conditions that may be contraindications for specific medicines - Current medication - Height and weight (measured and recorded against the normal range for age, height and sex) - Baseline pulse and blood pressure (measured with an appropriately sized cuff and compared with the normal range for age) - A cardiovascular assessment - An electrocardiogram (ECG) if the treatment may affect the QT interval. - Refer for a cardiology opinion before starting medication for ADHD if any of the following apply: - History of congenital heart disease or previous cardiac surgery - History of sudden death in a first-degree relative under 40 years suggesting a cardiac disease - Shortness of breath on exertion compared with peers - Fainting on exertion or in response to fright or noise - Palpitations that are rapid, regular and start and stop suddenly (fleeting occasional bumps are usually ectopic and do not need investigation) - Chest pain suggesting cardiac origin - Signs of heart failure - A murmur heard on cardiac examination - Blood pressure that is classified as hypertensive for adults - Refer to a paediatric hypertension specialist before starting medication for ADHD if blood pressure is consistently above the 95^th^ centile for age and height for children and young people.   **Prescribing Stimulants:** (MPH, LDX, DEX, ATX)  **Medication choice – children aged 5 years and over and young people:**   - Offer **MPH** (either short or long acting) as the first line pharmacological treatment for children aged 5 years and over and young people with ADHD. - Consider switching to **LDX** for children aged 5 years and over and young people who have had a 6-week trial of MPH at an adequate dose and not derived enough benefit in terms of reduced ADHD symptoms and associated impairment. - Consider DEX for children aged 5 years and over and young people whose ADHD symptoms are responding to LDX but who cannot tolerate the longer effect profile. - Offer ATX or GUA to children aged 5 years and over and young people if: - They cannot tolerate MPH or LDX or - Their symptoms have not responded to separate 6-week trials of LDX and MPH, having considered alternative preparations and adequate doses.   **Medication choice – adults:**   - **First-line:** Offer LDX or MPH as first-line pharmacological treatment for adults with ADHD. - Consider switching to LDX for adults who have had a 6-week trial of MPH at an adequate dose but have not derived enough benefit in terms of reduced ADHD symptoms and associated impairment. - Consider switching to MPH for adults who have had a 6-week trial of LDX at an adequate dose but have not derived enough benefit in terms of reduced ADHD symptoms and associated impairment. - Consider DEX for adults whose ADHD symptoms are responding to LDX but who cannot tolerate the longer effect profile. - Offer ATX to adults if: - They cannot tolerate LDX or MPH or - Their symptoms have not responded to separate 6-week trials of LDX and MPH, having considered alternative preparations and adequate doses.   **Further medication choices:**   - Obtain a second opinion or refer to a tertiary service if ADHD symptoms in a child aged 5 years or over, a young person or adult are unresponsive to one or more stimulants and one non-stimulant. - Do not offer any of the following medication for ADHD without advice from a tertiary ADHD service: - GUA for adults - Clonidine for children with ADHD and sleep disturbance, rages or tics - Atypical antipsychotics in addition to stimulants for people with ADHD and coexisting pervasive aggression, rages or irritability   **Medication choice – people with coexisting conditions:**   - Offer the same medication choices to people with ADHD and anxiety disorder, tic disorder or autism spectrum disorder as other people with ADHD. - For children aged 5 years and over, young people and adults with ADHD experiencing an acute psychotic or manic episode: - Stop any medication for ADHD - Consider restarting or starting new ADHD medication after the episode has resolved, taking into account the individual circumstances, risks and benefits of the ADHD medication.   *For further details on ‘considerations when prescribing ADHD medication’, ‘dose titration’, ‘shared care for medication’, please refer to the full NICE guidance.*  **Maintenance and monitoring:**  *For monitoring details including Height and weight, Cardiovascular, Tics, Sexual dysfunction, Seizures, Sleep, Worsening behaviour, Stimulant diversion, please refer to the full NICE guidance.*  **Adherence to treatment:**  *This items included ‘Supporting adherence to non-pharmacological treatments’ in the full NICE guidance.*  **Review of medication and discontinuation:**   - A healthcare professional with training and expertise in managing ADHD should review ADHD medication at least once a year and discuss with the person with ADHD (and their families and carers as appropriate) whether medication should be continued. The review should include a comprehensive assessment of the: - Preference of the child, young person or adult with ADHD (and their family or carers as appropriate) - Benefits, including how well the current treatment is working throughout the day - Adverse effects - Clinical need and whether medication has been optimised - Impact on education and employment - Effects of missed doses, planned dose reductions and periods of no treatment - Effect of medication on existing or new mental health, physical health or neurodevelopmental conditions - Need for support and type of support (for example, psychological, educational, and social) if medication has been optimised but ADHD symptoms continue to cause a significant impairment. - Encourage people with ADHD to discuss any preferences to stop or change medication and to be involved in any decisions about stopping treatments. - Consider trial periods of stopping medication or reducing the dose when assessment of the overall balance of benefits and harms suggests this may be appropriate. If the decision is made to continue medication, the reasons for this should be documented. |

**Abbreviations:** AAP: American Academy of Pediatrics, ATX: atomoxetine; CADDRA: Canadian ADHD Resource Alliance; DEX: dexamfetamine; GUA: guanfacine; LDX: lisdexamfetamine dimesylate; MPH: methylphenidate; NHMRC: National Health Medical Research Center ; NICE: National Institute of Health and Care Excellence ; SMOH: Singapore Ministry of Health ; UHMS: University of Michigan Health System
